# Influence of measurement differences of anterior chamber depth and axial length on lens thickness evaluation in cataract patients: a comparison of two tests

**DOI:** 10.1186/s12886-020-01754-w

**Published:** 2020-12-07

**Authors:** Jiayi Xu, Chen Li, Lijun Wang, Caixin Li, Xin Li, Peirong Lu

**Affiliations:** Department of Ophthalmology, the First Affiliated Hospital of Soochow University, 188 Shizi Street, Suzhou, 215006 China

**Keywords:** Axial length, Anterior chamber depth, Lens thickness

## Abstract

**Background:**

The purpose of this study was to investigate the agreement of lens thickness (LT) measurements made by contact A-scan ultrasonography and Lenstar LS900 as well as the influence of anterior chamber depth (ACD) and axial length (AL) measurement differences on LT measurement in cataract patients in the two techniques.

**Methods:**

1247 cataract patients (1247 eyes) participated in this retrospective cross-sectional study. Ocular biometric measurements were performed with Lenstar LS900 and A-scan ultrasonography respectively, and the measured results of AL, ACD and LT were compared using Pearson correlation coefficients (r) and Bland-Altman analyses.

**Results:**

Bland-Altman analyses showed poor agreement between the A-scan ultrasonography and Lenstar LS900 in measuring AL and ACD. The average difference of LT was 0.01 mm; the consistency limit was − 0.86 mm, 0.88 mm; and 95.27% of datapoints were within the 95% consistency limit. The consistency of LT measurements between the two techniques was poor for those subjects whose ACD or AL values were beyond the 95% consistency limit. Among the subjects whose AL or ACD values measured by A-scan ultrasonography were greater than those measured by Lenstar LS900, 93.33% of them were within the 95% consistency limit, suggesting that the consistency of LT measurement between the two techniques was poor. Of patients whose ACD or AL measured by A-scan ultrasonography were smaller than that of Lenstar LS900, 96.01% of them were within the 95% consistency limit.

**Conclusions:**

There was good agreement of the LT measurements between A-scan ultrasonography and Lenstar LS900, except for the axis deviating from the apparent axis during A-scan ultrasonography. If this error can be avoided, A-scan ultrasonography can replace Lenstar LS900 in LT measurement in cataract patients.

## Background

As cataract surgery entered the era of refractive surgery, the goal of the surgery has changed from simply the correction of blindness to accurate refractive correction, and precise preoperative ocular biometry is a requirement for refractive cataract surgery. In ocular biometry, axial length (AL), anterior chamber depth (ACD), and lens thickness (LT) are three important parameters involved in intraocular lens (IOL) power calculation. AL is required by the most commonly used formulas such as the SRK/T, Holladay 1, Hoffer Q, and Haigis, and the ACD is required by the Haigis formula [1–3].

In the past, LT measurement has attracted a certain attention in studies of the cause, development, and regulation mechanisms of ametropia and the pathogenesis of primary angle closure glaucoma, but it has not been emphasized in cataract-related biometrics, and it was not involved in the early IOL calculation formulas [4, 5]. However, with the development of fourth- and fifth-generation IOL calculation formulas, LT is gradually attracting much interest due to the introduction of the concept of effective lens position (ELP) [6]. The measurement of LT is mainly performed by ultrasonic (US) biometry and optical biometry. Optical biometry has become the mainstream method of LT measurement due to its high accuracy and simple operation. US biometry has the advantages of not being affected by iris tissue or optical-media opacity. Although many studies have focused on the consistency of AL and ACD measurements between the two technologies, few studies have paid attention to the agreement of the two methods on the LT measurement. The aim of this study was to compare measurements of LT as well as AL and ACD provided by US biometry and optical biometry based on optical low-coherence interferometry (OLCI). Furthermore, we analyzed the influence on the measurement of LT when there were large differences in the AL and ACD measurements made by the two technologies.

## Methods

This retrospective study was conducted at the First Affiliated Hospital of Soochow University, Suzhou, China from March 2016 to January 2020. Only preoperation cataract eyes were included, and only one eye of each patient was analyzed. Cataract diagnosis and grading were performed according to the Lens Opacities Classification System III (LOCS III). The degree of lens nucleus hardness was classified according to the Emery-Little classification, and only grades II-V were included in the study. Cases with very dense cataracts or subcapsular cataracts were excluded. The study protocol was approved by the institutional review boards of the First Affiliated Hospital of Soochow University and carried out in accordance with the tenets of the Declaration of Helsinki. Exclusion criteria included any history of intraocular surgery, corneal surgery, ocular trauma, corneal opacity, contact lens use, and those without complete data. The measurements were performed under the same lighting conditions by the same experienced ophthalmic technician [7].

AL, LT, and ACD were measured by a contact A-scan ultrasonography (UD-8000 + AL-4000, TOMY Inc., Tokyo, Japan) and optical biometry (Lenstar LS900, Haag-Streit AG, Koeniz, Switzerland). We set different US velocities for different tissues in the eye: the cornea and lens used 1641 m/sec, and the aqueous and vitreous used 1532 m/sec [8]. An average of 10 measurements were taken for analysis with the A-scan ultrasonography. When measured with the Lenstar LS900, each subject was measured three times to ensure reproducibility, and the average of those measurements was used. The eyes were undilated at the time of all the measurements. During the examination, biometry was first performed using the Lenstar followed by the contact A-scan ultrasonography.

### Statistical analysis

Bland-Altman plots were used to evaluate the agreement between OLCI and A-scan US measurements. The statistical analysis was performed with SPSS (ver 23.0). The statistical significance of the interdevice differences in ACD, AL, and LT measurements were evaluated with a paired two-tailed *t*-test. Agreement was evaluated using a 95% limit of agreement (LoA). The significance level for all tests was set at 5%. Pearson correlation coefficients (r) were used to investigate the relationship between the two instruments. *P* values of less than 0.05 were considered to be statistically significant.

## Results

**Fig. 1:**
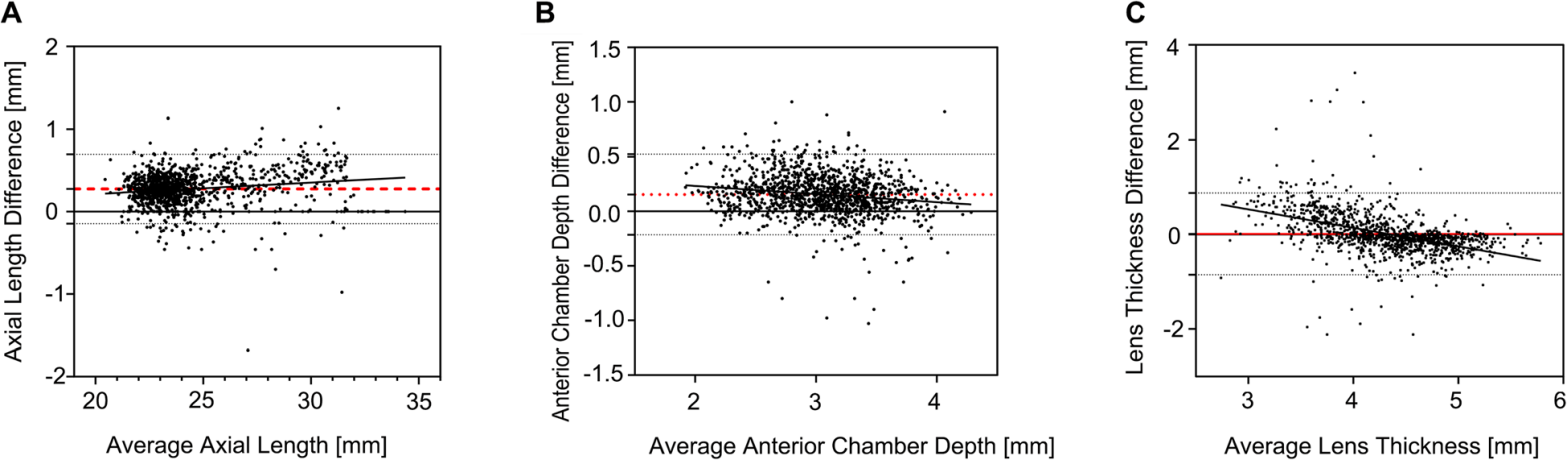
Bland-Altman plots for AL, ACD, and LT values between OLCI and US. **a** The Bland-Altman plots for AL between OLCI and US. **b** Bland-Altman plots for ACD between OLCI and US. **c** Bland-Altman plots for LT between OLCI and US. The red lines represent mean differences, and the dotted lines represent the 95% LoA values

A total of 1247 cataract patients were included in the study. The patients’ mean age was 67.39 years ±11.41 (SD) and 39.5% were male. The result of Pearson correlation analysis demonstrated a strong linear correlation between the OLCI and the US for AL, ACD, and LT measurements (Table 1). Table 2 shows the mean values and standard deviations of the parameters measured by the two technologies. A Bland-Altman consistency analysis showed that the mean difference in AL measured by the two instruments was 0.27 mm and that the OLCI method measured larger AL values compared with the US method. The consistency limit was − 0.15 mm, 0.70 mm, and 94.63% of datapoints were within the 95% consistency limit. The average difference between ACD measurements was 0.15 mm and the consistency limit was − 0.22 mm, 0.52 mm, with 94.95% of datapoints within the 95% consistency limit. These results showed poor agreement between the OLCI and the US in the measurement of AL and ACD (Fig. 1a, b). The average difference between LT measurements was 0.01 mm and the consistency limit was − 0.86 mm, 0.88 mm, with 95.27% of datapoints within the 95% consistency limit (Fig. 1c, Table 3). These results showed that although the two methods were not consistent in measuring AL and ACD, they were generally consistent in determining lens thickness.

**Table 1:** Pearson’s correlation coefficients (r) for the biometry obtained using the two technologies

Parameter	r	*P* value
AL (mm)	0.9965	< 0.0001
ACD (mm)	0.9101	< 0.0001
LT (mm)	0.7496	< 0.0001

*AL*  axial length; *ACD* anterior chamber depth (epithelium to the anterior lens surface); *LT* lens thickness; *OLCI* optical low-coherence interferometry; *US* ultrasound

**Table 2:** The mean values obtained by the two technologies

Parameter	OLCI	US	*P* value
AL (mm)	24.53 ± 2.56	24.25 ± 2.52	< 0.01
ACD (mm)	3.13 ± 0.42	2.97 ± 0.45	< 0.01
LT (mm)	4.33 ± 0.47	4.32 ± 0.67	0.6397

*AL* axial length; *ACD* anterior chamber depth (epithelium to the anterior lens surface); *LT* lens thickness; *OLCI* optical low-coherence interferometry; *US* ultrasound

**Table 3:** Agreement between OLCR and US in measuring AL, ACD and LT

Parameter	Mean differences	SD	95% limits of agreement
AL (mm)	0.27	0.21	−0.15 to 0.70
ACD (mm)	0.15	0.19	−0.22 to 0.52
LT (mm)	0.01	0.44	−0.86 to 0.88

**Fig. 2:**
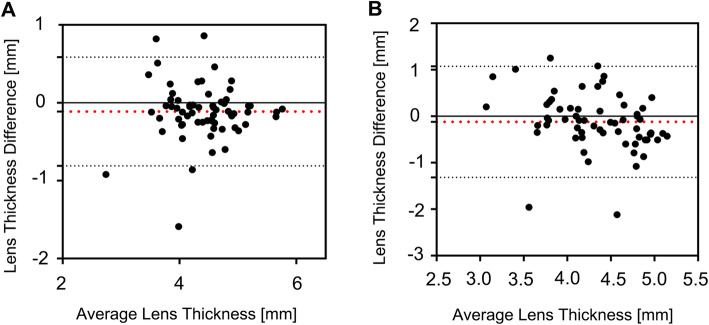
Bland-Altman plots for LT values of individuals whose AL and ACD values differed greatly for OLCI and US. **a** Bland-Altman plots for LT between OLCI and US of individuals whose AL values greatly differed. **b** Bland-Altman plots for LT between OLCI and US of individuals whose ACD values greatly differed between the two technologies. The red lines represent the mean differences, and the dotted lines represent the 95% LoA values

The results above showed that the overall consistency of the two detection methods in the measurement of AL and ACD was not so good. To observe whether the differences in AL and ACD measurements by the two methods will affect their measurement of LT, we analyzed the data from those subjects whose AL values were beyond the 95% consistency limit. Among those 67 cases, the mean difference in LT was − 0.11 mm and the consistency limit was − 0.81 mm, 0.59 mm, with 92.54% of datapoints within the 95% consistency limit (Fig. 2a). Following the same approach, the data from the 63 subjects whose ACD values were beyond the 95% consistency limit were also analyzed. The mean difference in LT was − 0.12 mm and the consistency limit was − 1.32 mm, 1.08 mm, with 93.65% of datapoints within the 95% consistency limit (Fig. 2b). These results indicated that when the differences between AL and ACD were large, the consistency of the Lenstar and A-Scan LT measurements was poor.

**Fig. 3:**
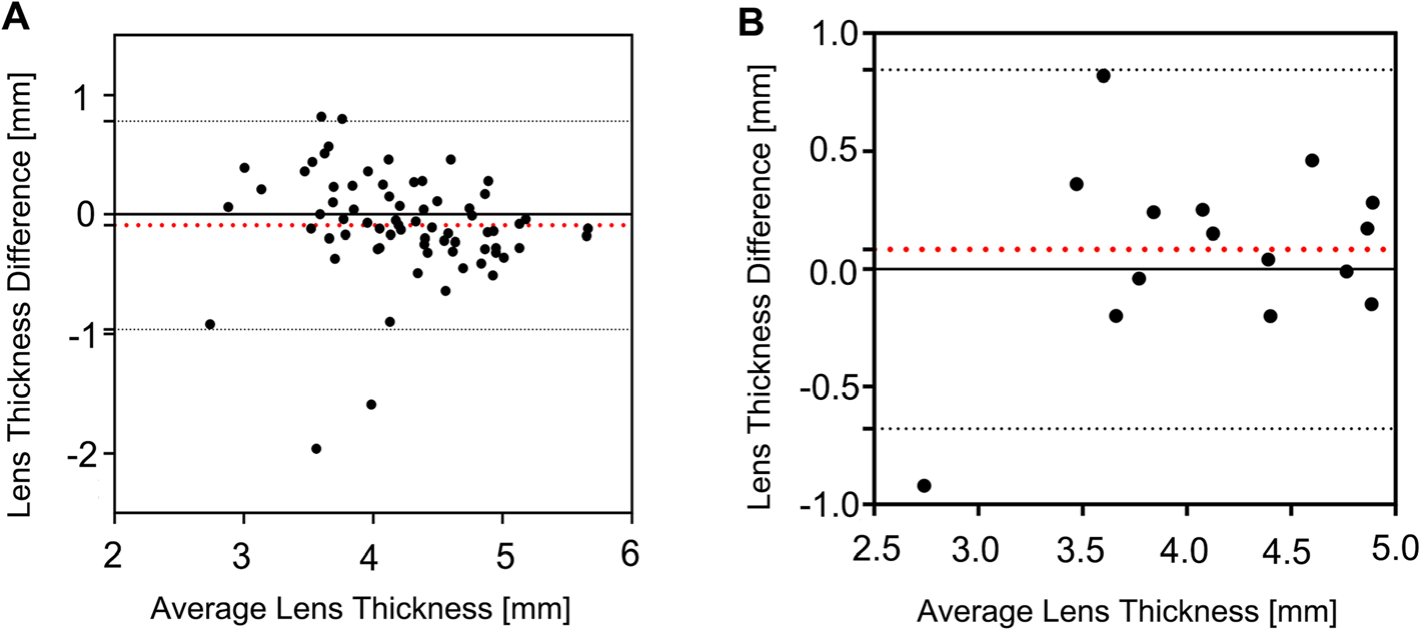
Bland-Altman plots for LT values between OLCI and US. AL_US_ was greater than that of AL_OLCI_ and ACD_US_ was greater than that of ACD_OLCI_. **a** Bland-Altman plots for LT between the two technologies when AL_US_ was greater than that of AL_OLCI_. **b** Bland-Altman plots for LT between the two technologies when ACD_US_ was greater than that of ACD_OLCI_. The red lines represent the mean differences, and the dotted lines represent the 95% LoA values

To further explore the potential causes of these differences and their effects on LT measurements, we extracted the data of 70 subjects whose AL values measured by US (AL_US_) were greater than that those measured by OLCI (AL_OLCI_). Of these patients, the mean difference in LT was − 0.09 mm and the consistency limit was − 0.96 mm, 0.78 mm, with 94.29% of datapoints within the 95% consistency limit (Fig. 3a). There were 15 patients whose ACD values measured by US (ACD_US_) were greater than those measured by OLCI (ACD_OLCI_). The mean difference in LT was 0.08 mm and the consistency limit was − 0.68 mm, 0.84 mm, with 93.33% of datapoints within the 95% consistency limit (Fig. 3b). As in the process of US measurement, the deviation of the measurement axis from the eye axis might cause the AL and the ACD measurements to be longer than they were in reality (Fig. 5c). Our results showed that the deviation of the measurement axis from the eye axis during US measurement was in fact what led to the inaccuracy of LT measurements (that is, those that were inconsistent with the Lenstar results).

**Fig. 4:**
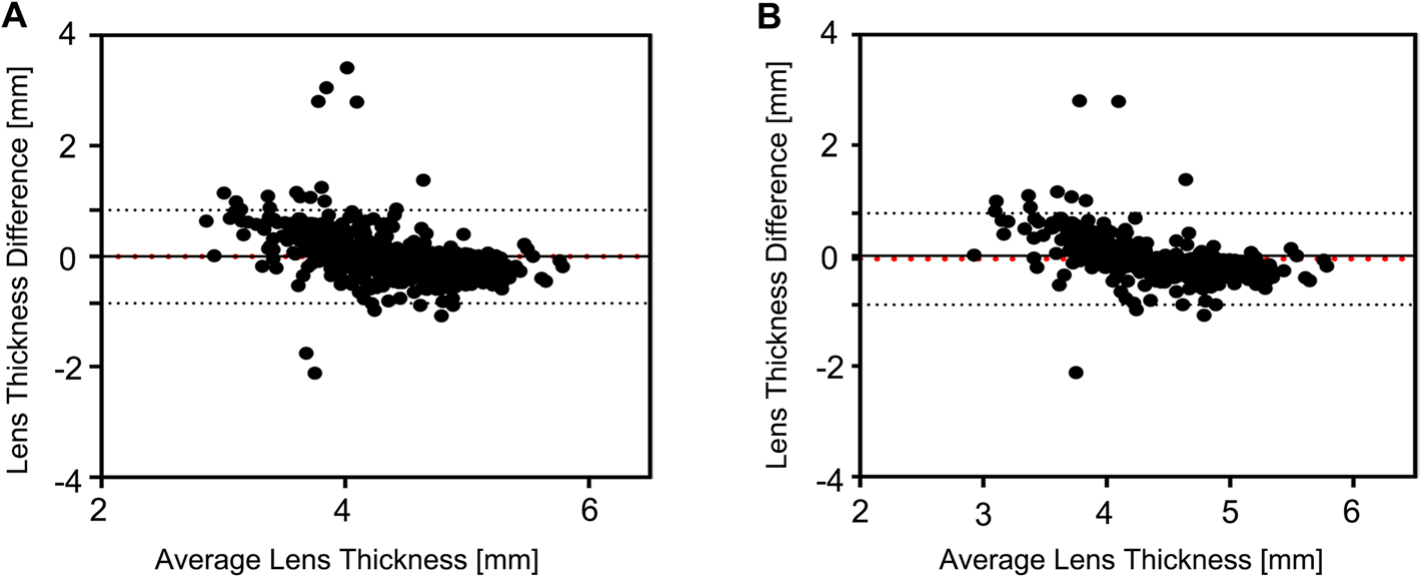
Bland-Altman plots for LT values between OLCI and US when AL_US_ was smaller than that of AL_OLCI_ and ACD_US_ was smaller than that of ACD_OLCI_. **a** Bland-Altman plots for LT values between the two technologies when AL_US_ was smaller than that of AL_OLCI_. **b** Bland-Altman plots for LT values between the two technologies when ACD_US_ was smaller than that of ACD_OLCI_. The red lines represent the mean differences, and the dotted lines represent the 95% LoA values

**Fig. 5:**
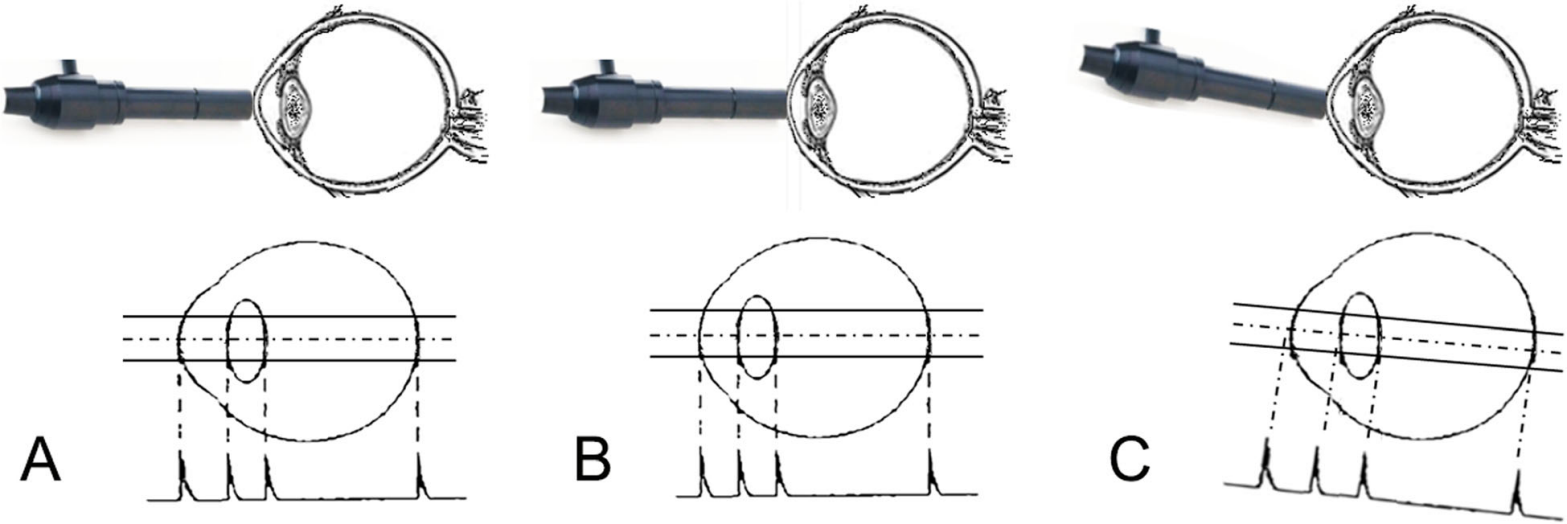
A schematic diagram of the ultrasonic eye biometric technique and its influence on LT measurement. **a** Correct measurement method. **b** When the probe presses on the eyeball, the measured ACD and AL will become shorter. **c** Deviation between the measurement axis and the eye axis will result in a longer measured ACD and AL

An additional condition was that AL_US_ was smaller than that AL_OLCI_, and when we selected 0.28 mm or more (mean AL_OLCI_ – mean AL_US_) as the inclusion criterion, 623 patients remained. The mean value of LT difference was − 0.01 mm and the consistency limit was − 0.85 mm, 0.84 mm, with 96.47% of patients within the 95% consistency limit (Fig. 4a). Of the 351 patients whose ACD_US_ was 0.16 mm or more (mean ACD_OLCI_ – mean ACD_US_) smaller than their ACD_OLCI_, the average difference in LT was − 0.06 mm and the consistency limit was − 0.89 mm, 0.77 mm, with 96.01% of datapoints within the 95% consistency limit (Fig. 4b). During the contact US operation, the pressure of the US probe on the eyeball often made the measurements smaller (Fig. 5b). Our results indicated that this error does not have a significant impact on the measurement of LT.

## Discussion

Accurately predicting the ELP after phacoemulsification is still difficult, but it plays an important role in reducing the postoperative refractive error. Measurement errors of AL, ACD and LT are important factors contributing to errors in ELP estimation [9]. In this paper, we compared the differences between Lenstar and A-scan ultrasonography in measuring the AL, ACD and LT of cataract patients, and analyzed the possible causes of errors when the US and the OLCI are inconsistent in measuring LT.

According to previous reports, there has been no significant statistical difference between Lenstar and A-scan US in the measurement of LT [10, 11]. In this study also found no significant statistical difference in the measurement of LT by the two different techniques. Bland-Altman analysis showed that the two techniques had good consistency in LT measurement, which was consistent with previous studies. It thus seems that the measurement of LT by these two methods could be clinically interchangeable [12]. For the measurement of AL and ACD, there were large differences between the two techniques. Bland-Altman analysis also showed that the agreement of AL and ACD measurements was poor, which was consistent with previous studies [13, 14]. The reason for this difference could be the difference in the detection principle of the two instruments. Lenstar, an optical biometry instrument based on the principle of low coherent-light reflection, uses lasers to reach the surface of each ocular structure along the visual axis that are then reflected back separately. After receiving the data, the detector analyzes and processes it. Lenstar can complete the biological measurement of the ocular structure and observe the anterior and posterior surfaces of the lens and the density of the lens clearly, with high resolution and many repetitions [14–16]. A-scan US is a traditional biological measurement method, mainly based on the echo reflection principle of ultrasound to obtain the measurement values of ocular tissues. The calculation methods of AL and ACD are also different between the two technologies. Lenstar uses the interference degree of light to take measurements and conduct linear processing, and calculated through an A-scan algorithm, while US used segmented sound velocity to measure each tissue.

In order to explore the effects on LT measurement of the difference between AL and ACD as measured by the US and the OLCI, we further studied the individuals beyond the 95% consistency limit of AL and ACD, and assessed their agreement in LT measurement by a Bland-Altman analysis. The results showed that when the difference of AL and ACD measured by the two techniques was large, the consistency of LT was poor. This difference has a certain influence on the calculation of IOLs because it would have led to a different IOL power selection when using a newer formula, which predicts the ELP based on LT, such as Olsen or Barrett Universal II formulas [17]. During the operation of the contact US, when the ultrasonic probe was tilted and not along the visual axis, a larger result was often obtained, while a smaller result was often due to the compression of the ultrasonic probe on the eyeball. Our results showed that when AL_US_ was greater than AL_OLCI_ or ACD _US_ was greater than ACD _OLCI_, Bland-Altman analysis showed a poor agreement of LT measurement, suggesting that the tilt of the ultrasonic probe can affect the measurement of AL, ACD and LT. When AL_US_ was smaller than AL_OLCI_ or ACD _US_ was smaller than ACD _OLCI_, Bland-Altman analysis showed that the measurement of LT had good consistency between the two techniques, suggesting that when the ultrasound probe pressed the eyeball, the measurement of AL and ACD would be affected, but the measurement of LT was not affected.

Compared with samples from related articles published earlier, our study had a larger sample size. Most of the previous studies only compared the consistency of AL, ACD, and LT measurements between different instruments, but few studies paid attention to individuals with poor consistency. Little literature has reported the impact on LT measurement when there were large differences in AL and ACD measurements taken by different instruments or on the influence of deviation during ultrasonic operations on LT measurements. However, the current study also has limitations, such as its exclusive focus on cataract patients, and the degree of opacification of cataract may induce a certain deviation in A-scan results.

## Conclusions

The results of measurement were significantly different between OLCI and US for AL and ACD. Although there was good consistency overall between the two methods in LT measurements, there was also poor consistency in LT measurements in patients with significant differences in AL or ACD measurements. When the measured value of US was larger than that of the optical method, it will lead to poor consistency between the two methods in measuring AL, ACD, and LT. These results suggest that probe tilt should be avoided as much as possible during the operation of contact A-scan US, as it may cause errors in the measurement of LT and result in larger errors in the application of some newer intraocular lens calculation formulas. Our results may help to correctly judge the accuracy and clinical application of A-scan ultrasonography for lens thickness measurement in cataract patients.

## Data Availability

The datasets analyzed during the current study are not publicly available for confidentiality reasons; nevertheless, the corresponding author will provide them on reasonable request.

## Abbreviations

LT: Lens thickness
ACD: Anterior chamber depth
AL: Axial length
IOL: Intraocular lens
ELP: Effective lens position
IOL: Intraocular lens
ELP: Effective lens position
LOCS III: Lens Opacities Classification System III
LoA: Limits of agreement
